# Citation Analysis of Iranian Journal of Basic Medical Sciences in ISI Web of Knowledge, Scopus, and Google Scholar

**Published:** 2013-10

**Authors:** Leili Zarifmahmoudi, Hamid Reza Kianifar, Ramin Sadeghi

**Affiliations:** ^1 ^Nuclear Medicine Research Center, Mashhad University of Medical Sciences, Mashhad, Iran

**Keywords:** Citation tracking, Google Scholar, Impact factor, Iranian Journal of Basic Medical Sciences, Scopus, Web of Science

## Abstract

***Objective(s):*** Citation tracking is an important method to analyze the scientific impact of journal articles and can be done through Scopus (SC), Google Scholar (GS), or ISI web of knowledge (WOS). In the current study, we analyzed the citations to 2011-2012 articles of Iranian Journal of Basic Medical Sciences (IJBMS) in these three resources.

***Material and Methods:*** The relevant data from SC, GS, and WOS official websites. Total number of citations, their overlap and unique citations of these three recourses were evaluated.

***Results:*** WOS and SC covered 100% and GS covered 97% of the IJBMS items. Totally, 37 articles were cited at least once in one of the studied resources. Total number of citations were 20, 30, and 59 in WOS, SC, and GS respectively. Forty citations of GS, 6 citation of SC, and 2 citations of WOS were unique.

***Conclusion: ***Every scientific resource has its own inaccuracies in providing citation analysis information. Citation analysis studies are better to be done each year to correct any inaccuracy as soon as possible. IJBMS has gained considerable scientific attention from wide range of high impact journals and through citation tracking method; this visibility can be traced more thoroughly.

## Introduction

Scientific literature quality evaluation is an interesting subject for researchers to find out the prominent journals of their field. Assessing the scientific productivity of researchers, scholarly influence of the journals, and individual works is done using different bibloimetric techniques. Citation analysis is one of the major biliometrics methods for quality evaluation of the journals by analyzing the citations of journals which includes counting the citations, considering the citing items, and comparing citations distribution in different scientific indexing databases (1, 2). 

Citation analysis information used to be provided only by Web of Science (WOS) from ISI as the strongest scientific indexing database for years but since 2004, Scopus (SC) from Elsevier and Google Scholar (GS) from Google became two major resources for retrieving citation information in addition to WOS (3). Due to several differences among the three mentioned sources regarding their coverage, accessibility, updating half life, etc; all the mentioned sources should be considered while analyzing the citations of individual journals (4, 5).

Iranian Journal of Basic Medical Sciences (IJBMS) is one of the leading Iranian journals in basic medical sciences which publish literature on anatomical sciences, biochemistry, genetics, immunology, microbiology, pathology, pharmacology, pharmaceutical sciences, and physiology. 

IJBMS is indexed in ISI, SC, and GS and in 2011 received its first impact factor of 0.324 which is fairly high. In the current study we aimed to examine the citation analysis of IJBMS in these three scientific resources (WOS, SC, and GS), and to compare the discrepancies between their citation frequencies, and distribution. 

## Material and Methods

We considered all IJBMS articles published in 2011 and 2012. Citations to each article were extracted from all scientific databases and considered separately. Citations referred to all articles indexed in WOS, Scopus, and Google Scholar were extracted from their official website (-) using “cited by” feature for each article. 

**Table 1:** Details of the citations to Iranian Journal of Basic Medical Sciences articles in Scopus, ISI web of science, and Google scholar

Title	Citations in WOS	Citations in SC	Citations in GS	SC/WOS overlaps	SC/WOS overlaps	WOS/GS overlaps
Lectin Histochemical Study of Vasculogenesis During Rat Pituitary Morphogenesis	3	4	9	3	3	2
"Silymarin", a Promising Pharmacological Agent for Treatment of Diseases	3	2	0	2	2	0
The Effect of Linear PEI on Characteristics and Transfection Efficiency of PEI-Based Cationic Nanoliposomes	2	2	2	2	2	2
Plasma Nitric Oxide and Acute Phase Proteins after Moderate and Prolonged Exercises	1	1	1	1	1	1
Effects of Low-dose Morphine on Nitric Oxide Concentration and Angiogenesis in Two-kidney One Clip Hypertensive Rats	1	2	2	1	1	1
Bioactivity of Malva Sylvestris L., a Medicinal Plant from Iran	1	0	1	0	0	1
Combination of Stem Cell Mobilized by Granulocyte-Colony Stimulating Factor and Human Umbilical Cord Matrix Stem Cell: Therapy of Traumatic Brain Injury in Rats	1	1	1	1	1	1
Anxiolytic-like Effect of Testosterone in Male Rats: GARA© Receptors Are Not Involved	1	1	1	1	1	1
Profile of Iranian GJB2 Mutations in Young Population with Novel Mutation	1	1	2	0	0	0
Rosmarinic Acid Ameliorates Diabetic Nephropathy in Uninephrectomized Diabetic Rats	1	1	6	0	0	1
Effect of Aqueous-Ethanolic extract from Rosa damascena on Guinea Pig Isolated Heart	1	1	0	1	1	0
The Laxative and Prokinetic Effects of Rosa damascena Mill in Rats	1	1	1	1	1	0
Effect of Curcumin on Doxorubicin-induced Cytotoxicity in H9c2 Cardiomyoblast Cells	1	2	1	1	1	0
Effects of Chronic Oral Administration of Natural Honey on Ischemia/Reperfusion-induced Arrhythmias in Isolated Rat Heart	1	1	4	1	1	1
Identification of Nontuberculous Mycobacteria Species Isolated from Water Samples Using Phenotypic and Molecular Methods and Determination of their Antibiotic Resistance Patterns by E-Test Method, in Isfahan, Iran	0	2	0	0	0	0
Qnr Prevalence in Extended Spectrum Beta-Lactamases (ESBLs) and None- ESBLs Producing Escherichia coli Isolated fromUrinary Tract Infections in Central of Iran	0	2	0	0	0	0
Pharmacological Effects of Rosa damascena Effect of Matricaria Aurea (Loefl.) Shultz-Bip. Hydroalcoholic	0	2	7	0	0	0
Extract on Acetic Acid-induced Acute Colitis in Rats	0	2	1	0	0	0
Antileishmanial Activity of Liposomal Clarithromycin gainst Leishmania major Promastigotes	0	1	1	1	1	0
Effect of Hydroalcoholic and Buthanolic Extract of Cucumis Sativus Seeds on Blood Glucose Level of Normal and Streptozotocin-induced Diabetic Rats	0	1	1	0	0	0
Effect of Ethanolic Extract of Indigofera Tinctoria on Chemically-Induced Seizures and Brain GABA Levels in Albino Rats	0	1	0	0	0	0
Effects of Maternal Lead Acetate Exposure During Lactation on Postnatal Development of Testis in Offspring Wistar Rats	0	1	1	0	0	0
Effects of Infantile Repeated Hyperglycemia on Neuronal Density of Hippocampus and Pentylentetrazol Induced Convulsions in Male Wistar Rats	0	0	1	0	0	0
The Effect of Butter Oil on Avoidance Memory in Normal and Diabetic Rats	0	0	1	0	0	0
Endocannabinoid System and TRPV1 Receptors in the Dorsal Hippocampus of the Rats Modulate Anxiety-like Behaviors	0	0	1	0	0	0
Effects of Peripheral and Intra-Hippocampal Administration of Sodium Salicylate on Spatial Learning and Memory of Rats	0	0	1	0	0	0
Identification and Characterization of a High Vancomycin-Resistant Staphylococcus aureus Harboring VanA Gene Cluster Isolated from Diabetic Foot Ulcer	0	0	2	0	0	0
Different Expression of Extracellular Signal-regulated Kinases (ERK) 1/2 and Phospho-Erk Proteins in MBA-MB-231 and MCF-7 Cells After Chemotherapy with Doxorubicin or Docetaxel	0	0	1	0	0	0
Preparation, Characterization and Evaluation of Sun Protective and Moisturizing Effects of Nanoliposomes Containing Safranal	0	0	2	0	0	0
The Role of The Endocannabinoids in Suppression of The Hypothalamic-Pituitary-Adrenal Axis Activity By Doxepin	0	0	1	0	0	0
Synthesis of Novel 4-[1-(4-fluorobenzyl)-5-imidazolyl] Dihydropyridines and Studying their Effects on Rat Blood Pressure	0	0	1	0	0	0
Containing High Amount of Rate Retarding Eudragit Rl Using Peg 400 And Investigation of their Physicomechanical Properties						
Effects of Combined Sonodynamic and Photodynamic Therapies on a Colon Carcinoma Tumor Model	0	0	1	0	0	0
Comparing Effects of Aerobics, Pilates Exercises and Low Calorie Diet on Leptin Levels and Lipid Profiles in Sedentary Women	0	0	1	0	0	0
Prognostic Significance of MMP2 and MMP9 Functional Promoter Single Nucleotide Polymorphisms in Head and Neck Squamous Cell Carcinoma	0	0	1	0	0	0
The Effect of Chronic Administration of Aegle Marmelos Seed Extract on Learning and Memory in Diabetic Rats	0	0	1	0	0	0
Design of Agglomerated Crystals of Ibuprofen During Crystallization: Influence of Surfactant	0	0	1	0	0	0

**Table 2:** Journals citing Iranian Journal of Basic Medical Sciences and their 2011 impact factor. The information was retrieved from ISI web of knowledge official website

Impact	Citing Journal	All Yrs
	All Journals	59
0.324	IRAN J BASIC MED SCI	17
	ALL OTHERS (14)	14
0.988	INDIAN J MED MICROBI	2
1.389	J NAT MED-TOKYO	2
	J MED PLANTS RES	2
2.656	FOOD CONTROL	2
0.371	IRAN RED CRESCENT ME	1
0.421	IRAN J PARASITOL	1
2.823	J AGR FOOD CHEM	1
2.598	INT J MOL SCI	1
4.601	INORG CHEM	1
1.295	INDIAN J EXP BIOL	1
0.435	HEALTHMED	1
2.999	FOOD CHEM TOXICOL	1
1.061	EXCLI J	1
0.535	ACTA NEUROL BELG	1
0.176	ADV CLIN EXP MED	1
	ADV MATER RES-SWITZ	1
3.417	BEHAV BRAIN RES	1
	CELL J	1
1.804	CHEM BIODIVERS	1
1.776	CURR NANOSCI	1
0.625	DARU	1
1.082	DRUG CHEM TOXICOL	1
4.046	EUR NEUROPSYCHOPHARM	1
1.432	AAPS PHARMSCITECH	1

Coverage of databases, citation counts, and type of citing items were evaluated in each scientific database. The reasons of dissimilarity between IJBMS citation analyses of databases were evaluated in depth. Finally journals citing articles of IJBMS were analyzed according to their citation frequencies and 2011 Impact Factors (IF).

All information was retrieved on 10/2/2013 from the corresponding websites of the three mentioned resources (WOS, SC, GS). 

## Results

Overall 157 articles were published in IJBMS during 2011 and 2012. All of these articles could be located using WOS, and SC (100% database coverage). On the other hand GS had 97% coverage (5 articles could not be located by GS). 

**Figure 1:**
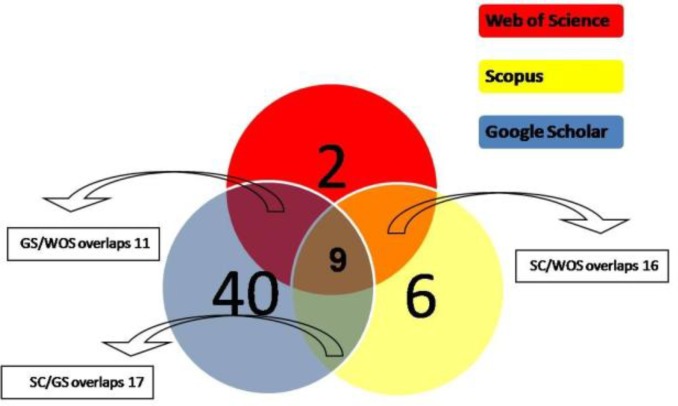
Citations to 2011 and 2012 Iranian Journal of Basic Medical Sciences articles and their overlaps in Scopus, google scholar, and web of science.

Totally, 37 articles were cited at least once in one of the studied databases (Table 1). Total number of citations was 20, 30, and 59 in WOS, SC, and GS respectively. Figure 1 shows the overlap and unique citations of each of the resources. Table 1 shows the details about citation analysis of IJBMS articles in the three WOS, GS, and SC. Table 2 shows the journals citing IJBMS articles in WOS and their Impact Factors (IF).

## Discussion

For years, ISI form Web of Knowledge was the only scientific database for retrieving citation analysis information. Scopus and Google Scholar are two major additions to the citation analysis which can be considered as WOS rivals. Unlike WOS and SC, GS is a free extensive search engine. There is a considerable uncertainty regarding GS journal coverage, despite covering most of the journals and not-English articles (3). The major reason of the different citation counts in each scientific database was differences in journal coverage of each database. In our study the citation count of IJBMS resulted in different citation counts in three studied resources (GS: 59, SC: 30, and WOS: 20) and the highest citation belonged to GS. There were citations from journal articles not indexed in SC or ISI, but covered in GS. Different journal coverage of each database results in some unique citations for each scientific source too. In our citation analysis, 40 citations of GS, 6 citation of SC, and 2 citations of WOS were unique.

Type of the citing items also causes different citation analysis results. Books, meeting abstracts, conference papers, and doctoral and master’s theses are usually considered only in GS citation counts (9). A study of IJBMS was cited by a proceeding paper which could be located by GS and SC but not by WOS (10). 

Upgrading intervals of the databases is a factor that may cause different citation analysis results in specific time points. Scientific databases have different upgrading strategies and yield different citation counts at each time point. Some citations may be located in GS which are not included in ISI or SC yet. Almost 15% of GS citations were articles published in 2013 and were not included in SC, or ISI, at the time our data were extracted. 

Inaccuracies of the databases in identifying the citations are another factor which resulted in different citation counts in the GS, WOS, and SC. Three articles of IJBMS showed discrepancies of citation counts of GS and WOS due to this fact. These citations could not be identified by WOS due to inaccurate indexing in WOS (-). Upon identification of these citations, we asked WOS to correct the inaccuracies which will be done in the upcoming months. A simple calculation shows that 2012 impact factor (IF) of IJBMS (which will be published in July 2013) actually increased by 0.026 (3/115; 115 is the number of IJBMS articles published in 2010 and 2011) through these corrections. This increase in IF shows the importance of citation analysis and we recommend citation tracking of IJBMS each year for detecting any WOS inaccuracies. 

Language coverage is another difference between scientific databases. Most of the journals indexed in WOS and SC are English language journals, but GS covers many not-English language journal articles. In our study there were two citations from Russian and Spanish articles which were not indexed by WOS and SC, but GS counted them as citing items. 

As shown in Table 2, IJBMS has been cited by variety of WOS indexed journals with impact factor as high as 4. This shows that IJBMS has got considerable visibility in scholarly world and has attracted the scientific researchers. Hopefully in the future this visibility would increase even more.

## Conclusion

Every scientific resource has its own inaccuracies in providing citation analysis information. Citation analysis studies are better to be done each year to correct any inaccuracy as soon as possible. IJBMS has gained considerable scientific attention from wide range of high impact journals and through citation tracking method; this visibility can be traced more thoroughly. 
